# The Essential Role of Tick Salivary Glands and Saliva in Tick Feeding and Pathogen Transmission

**DOI:** 10.3389/fcimb.2017.00281

**Published:** 2017-06-22

**Authors:** Ladislav Šimo, Maria Kazimirova, Jennifer Richardson, Sarah I. Bonnet

**Affiliations:** ^1^UMR BIPAR, INRA, Ecole Nationale Vétérinaire d'Alfort, ANSES, Université Paris-EstMaisons-Alfort, France; ^2^Institute of Zoology, Slovak Academy of SciencesBratislava, Slovakia; ^3^UMR Virologie, INRA, Ecole Nationale Vétérinaire d'Alfort, ANSES, Université Paris-EstMaisons-Alfort, France

**Keywords:** ticks, tick saliva, tick-borne pathogens, tick salivary glands

## Abstract

As long-term pool feeders, ticks have developed myriad strategies to remain discreetly but solidly attached to their hosts for the duration of their blood meal. The critical biological material that dampens host defenses and facilitates the flow of blood—thus assuring adequate feeding—is tick saliva. Saliva exhibits cytolytic, vasodilator, anticoagulant, anti-inflammatory, and immunosuppressive activity. This essential fluid is secreted by the salivary glands, which also mediate several other biological functions, including secretion of cement and hygroscopic components, as well as the watery component of blood as regards hard ticks. When salivary glands are invaded by tick-borne pathogens, pathogens may be transmitted via saliva, which is injected alternately with blood uptake during the tick bite. Both salivary glands and saliva thus play a key role in transmission of pathogenic microorganisms to vertebrate hosts. During their long co-evolution with ticks and vertebrate hosts, microorganisms have indeed developed various strategies to exploit tick salivary molecules to ensure both acquisition by ticks and transmission, local infection and systemic dissemination within the vertebrate host.

## Introduction

Ticks are obligate hematophagous arthropods and act as vectors of the greatest variety of pathogens including viruses, parasites, and bacteria (de la Fuente et al., 2008; Rizzoli et al., 2014). On a global scale, they represent the most important vectors of pathogens that affect animals, and are second only to mosquitoes where humans are concerned (Dantas-Torres et al., 2012). Their remarkable success as disease vectors is mainly related to their longevity, high reproductive potential and broad host spectrum for several species, as well as to their capacity to imbibe a very large quantity of blood over a relatively long period of time.

**Figure 1:**
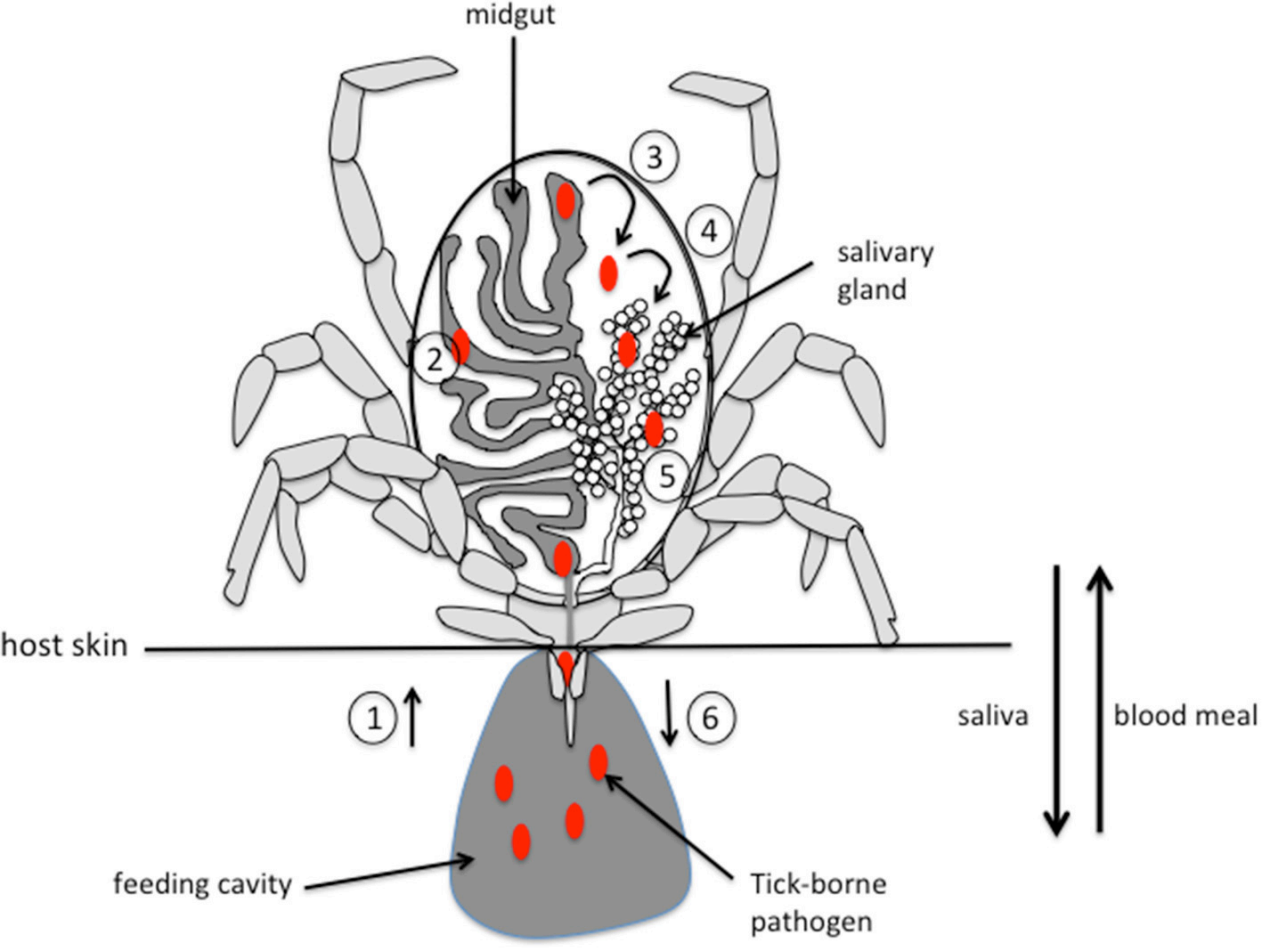
Schematic representation of pathogen acquisition, development and transmission by a tick. (1) pathogens are ingested by the tick along with the blood meal during the bite. (2) Pathogens invade the midgut and, depending on the species, stay in the midgut until the next feeding or immediately cross the epithelium of the digestive tract (3) to invade the tick body. (4) Pathogens move into the salivary glands by crossing the epithelium and invade the acini (5). (6) Pathogens are injected into a new host during feeding, along with saliva that counteracts host hemostasis, inflammation and immune responses, thus facilitating pathogen infection of host. Please note that, for clarity, only half of the digestive tract and a single salivary gland are represented.

For most tick-borne pathogens (TBP), transmission to the vertebrate host occurs via the saliva, underscoring the importance of both salivary glands (SG) and saliva in the transmission process. During feeding, ticks inject saliva and absorb their meal in an alternating pattern through the same canal. They are pool feeders, ingurgitating all of the fluids that are exuded into the haemorrhagic pool generated by the bite. TBP are ingested by ticks during their feeding on infected hosts. From the midgut, TBP cross the digestive epithelium and invade the haemocoel, from which they can penetrate the SG epithelium to invade the SG. From there, TBP can be transmitted to a new host via saliva injected during a new blood meal (Figure 1).

Vertebrates react to skin injury inflicted by tick bites by the formation of a haemostatic plug, vasoconstriction, inflammation and tissue remodeling related to wound healing. If unchecked, these processes would cause tick rejection and/or disrupt tick feeding, and arrest their further development. To facilitate the flow of blood and assure feeding, however, ticks have evolved a complex and sophisticated pharmacological armament that blocks pain and itch, inhibits haemostasis, and modulates innate and adaptive immune responses, angiogenesis and wound healing in their hosts (Francischetti et al., 2009; Mans, 2011; Kazimirova and Stibraniova, 2013; Štibrániová et al., 2013; Wikel, 2013; Valdes, 2014; Kotal et al., 2015; Chmelar et al., 2016b). It has been demonstrated that these molecules create a favorable environment for transmission, survival, and propagation of TBP within the vertebrate host (Wikel, 1999; Kazimirova and Stibraniova, 2013). In addition, several studies have reported that tick SG differentially express transcripts and proteins in response to pathogen infection, but only a few SG factors have been identified as directly implicated in pathogen transmission (Liu and Bonnet, 2014). In this review, we will summarize the essential role of both SG and saliva in tick biology as well as in TBP acquisition and transmission.

## Tick salivary glands

The tick SG play multiple essential functions during both on- and off-host periods and represent a key route in transmission of TBP. The physiological activity and unique morphology of this tissue are intimately associated with adaptation of the tick to the parasitic lifestyle. Here we briefly describe the structure and function of SG in both ixodid and argasid ticks and discuss the most important findings with respect to the physiology of SG secretion in ixodid ticks.

### Structure and function of salivary glands

In both argasid and ixodid ticks the female SG consist of a large number of acini (or otherwise called alveoli) of three different types (type I, II, and III) in ixodid and two different types (type I and II) in argasid ticks. In addition, ixodid males possess a fourth type (type IV) of acini in their SG. Agranular type I acini connect almost exclusively to the anterior part of the main salivary duct, while the granular type II and III acini are associated with more distally located secondary and tertiary ducts, respectively (Coons and Roshdy, 1973; Binnington, 1978; Fawcett et al., 1981a; Walker et al., 1985; Sonenshine, 1991). The agranular acini are morphologicaly similar in both argasid and ixodid ticks, and generally comprise four distinct cell types: a single central lamellate cell, multiple peripheral lamellate cells, peritubular cells, and one circumlumenal cell (Needham et al., 1990; Sonenshine, 1991). In type II and III acini, in addition to various agranular cell types (such as epithelial, adlumenal, ablumenal interstitial, and neck cells), 7–9 various glandular cells (divided into the *a-f* types depending on tick species), enclosing the secretory granules have generally been recognized (Fawcett et al., 1986; Sonenshine, 1991). The single adlumenal cell, also called the Cap or myoepithelial cell (Meredith and Kaufman, 1973; Krolak et al., 1982), lines the luminal surface of the type II and III acini in web-like fashion, and its contractions facilitate expulsion of the acinar contents into the connecting ducts during tick feeding (Krolak et al., 1982; Coons et al., 1994; Šimo et al., 2014b). During feeding of Ixodid females, the majority of the acinar cells of both type II and III acini undergo marked hypertrophy, resulting in overall increase in the mass of the SG (Binnington, 1978; Fawcett et al., 1986; Šimo et al., 2013). In particular, the lumen of type III acini greatly expands due to fluid uptake from the hemolymph (Meredith and Kaufman, 1973; Fawcett et al., 1981a; Kim et al., 2014), while in type II acini the cell bodies enlarge and the lumen remains proportionally smaller (Binnington, 1978; Walker et al., 1985).

Tick SG mediate diverse functions that ensure the tick's biological success during both on- and off-host periods. Here, we briefly discuss their role in absorption of moisture from unsaturated atmosphere, concentration of the nutrient portion of the blood meal by elimination of excess fluid and, finally, production of the cement that anchors the hypostome in the host skin. The crucial role of tick SG in secretion of biologically active molecules that facilitate acquisition of the blood meal and TBP development is elaborated later in this review.

During the fasting period off the host, the conservation of water is critical for ticks to avoid death due to desiccation. It is generally believed that the type I acini are actively involved in absorption of humidity from the surrounding environment. Under desiccating conditions these structures secrete a highly hygroscopic solution rich in Na^+^, K^+^, and Cl^−^ onto the surface of the mouthparts, which is subsequently swallowed back along with absorbed moisture (Knulle and Rudolph, 1982; Needham and Teel, 1986; Needham et al., 1990; Sonenshine, 1991; Gaede and Knülle, 1997). The absorptive property of type I acini was recently confirmed by an elegant experiment in which a fluorescent trace dye (Rhodamine 123) imbibed by desiccated *I. scapularis* females was shown to accumulate exclusively in type I acini (Kim et al., 2016).

Among blood-feeding arthropods, ixodid ticks are unique in the long duration of attachment to the host, which varies from several days up to weeks depending on life stage and tick species. When feeding, female ticks are capable of increasing their weight more than 100-fold due to the blood-meal uptake. At the same time, a large amount of excess fluid (including ions) is excreted back to the feeding site via SG, thus maintaining homeostasis (Balashov, 1972; Sonenshine, 1991; Sauer et al., 1995). In particular, water and ions from the digested blood meal cross the wall of the midgut into the hemocoel where they are taken up by SG and subsequently secreted via the salivary ducts back into the host. In 1973, Kaufman and Philips reported that in *Dermacentor andersoni* females 74% of the water and 96% of the sodium are expelled back to the host via this route. Several studies have suggested that the epithelium of type III acini is the site at which water and electrolytes from surrounding haemolymph gain access into SG (Meredith and Kaufman, 1973; Fawcett et al., 1981b). This hypothesis has been reinforced by a recent study in which the sodium-potassium pump (Na/K-ATPase) involved in formation of the sodium-rich primary saliva was evidenced in the epithelial cells of all three types of SG acini (Kim et al., 2016). In argasid ticks, the mechanism by which excess water is eliminated is different, being accomplished by the coxal glands, which are unique to this tick family (Binnington, 1975).

When attached to a host, the mouthparts of most of the ixodid ticks are encased by a cement cone, allowing the ticks to anchor themselves firmly in the host skin and simultaneously protecting the mouthparts from the host immune system (Kemp et al., 1982). The production of the cement cone has been observed exclusively in the ixodid lineage, although not all of the species from the *Ixodes* genus produce cement (Kemp et al., 1982; Mans, 2014). The origin of this substance, composed of polymerized and hardened glycine-rich proteins, lipids and certain carbohydrates, are the cells of the type II and III SG acini (Chinery, 1973; Jaworski et al., 1992). In addition to glycine-rich proteins, recent proteomic analyses of the cement cone from *Amblyomma americanum* evidenced multiple serine protease inhibitors and metalloproteases (Bullard et al., 2016), some of them considered to be promising anti-tick vaccine candidates due to their antigenic properties (Shapiro et al., 1987; Mulenga et al., 1999; Bishop et al., 2002).

### Physiology of salivary gland secretion

The tick SG is controlled by the nerves arising from the synganglion, the central nervous system of ticks (Kaufman and Harris, 1983; Fawcett et al., 1986; Šimo et al., 2009a,b, 2012, 2014a,b). Over the past four decades, pharmacological studies have revealed multiple chemical agents capable of inducing—either directly or indirectly—tick SG secretion (Kaufman, 1978; Sauer et al., 1995, 2000; Bowman and Sauer, 2004; Kim et al., 2014). Among these, catecholamines have been shown to be particularly effective activators of SG fluid secretion in both *in vivo* and *in vitro* assays (Kaufman, 1976; Lindsay and Kaufman, 1986; McSwain et al., 1992; Šimo et al., 2012). The downstream action of dopamine, the most potent activator of SG fluid secretion, has been studied in detail. It has been suggested that dopamine autocrine/paracrine signaling in tick SG (Kaufman, 1977, 1978; Šimo et al., 2011; Koči et al., 2014) activates two independent downstream pathways: cAMP-dependent transduction, which results in fluid secretion, and a calcium-dependent pathway leading to the secretion of prostaglandin E_2_ (PGE_2_) into the salivary cocktail. PGE_2_ may subsequently induce the exocytosis of anticoagulant proteins via paracrine signal in tick SG (Qian et al., 1998; Sauer et al., 2000). Dopamine acts via two different cognate receptors expressed in both type II and III SG acini: the D1 dopamine receptor and the invertebrate-specific D1-like dopamine receptor (InvD1L). Pharmacological study of these receptors has revealed that activation of the D1 receptor preferentially triggers the cAMP-dependent downstream pathway, while activation of the InvD1L exclusively causes mobilization of intracellular calcium (Šimo et al., 2011, 2014b). Based on immunohistochemical studies and subsequent physiological experiments performed on isolated SG, distinct structure-function relationships have been proposed for each of these receptors. In particular, the D1 receptor, which has been localized in cell junctions on the luminal surface of type II and III acini, may regulate the inward transport of fluid into the acini, while the InvD1L receptor, found to be expressed in the axon terminals in proximity to the myoepithelial cell in type II and III acini, is presumed to be involved in the expulsion of the acinar context into the connecting ducts (Šimo et al., 2011, 2014b; Kim et al., 2014).

In addition to dopamine, several other pharmacological agents, such as octopamine, norepinephrine, γ-aminobutyric acid (GABA), ergot alkaloids and pilocarpine, have been shown to exert either a direct or indirect effect on salivary secretion; their precise mode of action, however, remains enigmatic (Needham and Pannabecker, 1983; Pannabecker and Needham, 1985; Lindsay and Kaufman, 1986).

Based on recent studies, it appears that ticks are capable of selectively controlling particular types of acini (and likely individual cells within the acini) via the neuropeptidergic network arising from their synganglion and in which the SG is connected (Šimo et al., 2009a,b, 2012, 2013, 2014a; Roller et al., 2015). In particular, two neuropeptides, myoinhibitory peptide (MIP) and SIFamide, have been found to be co-expressed in the pair of giant neurons in the synganglion whose axonal projections reach the basal regions of type II and III acini (Šimo et al., 2009a,b, 2011, 2012, 2013, 2014a). The SIFamide receptor, as evidenced by immunostaining, was found in proximity to the acinar valve (basal region of acini), suggesting its role in control of this structure (Šimo et al., 2009b, 2013). Using immunohistochemical approaches, two other putative neuropeptides, orcokinin and pigment dispersing factor (PDF), were found in two pairs of neurons innervating exclusively type II SG acini (Šimo et al., 2009a, 2012; Roller et al., 2015). In summary, multiple axonal projections, expressing diverse signaling molecules or their receptors, reach the individual SG acini and regulate a variety of essential processes, presumably in response to the tick's fluctuating physiological requirements.

## Tick saliva

To understand the complex feeding biology of ticks, as well as the transmission of TBP, the composition of tick saliva must be molecularly resolved. Such resolution would underpin discovery of pharmacologically active compounds of clinical interest, protective antigens for anti-tick vaccines and antigens whose cognate antibody responses represent serological biomarkers of exposure to ticks. The first proteomic studies that addressed tick saliva date back to the first decade of the twenty-first century (Madden et al., 2004; Narasimhan et al., 2007a; Francischetti et al., 2008). Since then, transcript and protein profiling in tick SG have been applied to different developmental stages, sexes, and feeding stages of several species of hard and soft ticks. In addition, comparative analyses of tick SG have revealed that molecular expression varied according to tick life stage, sex, or behavior (Ribeiro et al., 2006; Anatriello et al., 2010; Diaz-Martin et al., 2013b), as well as according to the presence of pathogenic microorganism (Liu and Bonnet, 2014). It should be noted, however, that a minority of the salivary proteins have been functionally annotated, and that of these, the putative function has been verified for fewer than 5% (Francischetti et al., 2009). Nevertheless, these studies have already led to the discovery of multiple factors that contribute to successful tick feeding and evasion of the host immune and haemostatic defenses, which are reviewed below and presented in Table 1.

**Table 1:** Identified salivary tick molecules involved in modulation of host defense responses.

**Target mechanism**	**Molecule**	**Tick species**	**References**
***Vasodilation/Vasoconstriction***	Prostacyclin	*Ixodes scapularis*	Ribeiro and Mather, 1998
	tHRF	*I. scapularis*	Dai et al., 2010
	Prostaglandins	*Amblyomma americanum*	Bowman et al., 1996
***Wound healing/Angiogenesis***	Metalloproteases	*Ixodes ricinus*	Decrem et al., 2008a
	Metalloproteases	*I. scapularis*	Francischetti et al., 2005a
	ISL 929, ISL 1373	*I. scapularis*	Guo et al., 2009
	Haemangin	*Haemaphysalis longicornis*	Islam et al., 2009
	HLTnI	*H. longicornis*	Fukumoto et al., 2006
	PGE_2_	*Dermacentor variabilis*	Poole et al., 2013a,b
***Platelet aggregation***	Apyrase	*Ornithodoros* spp. *I. scapularis*	Ribeiro et al., 1985; Mans et al., 1998a
	Moubatin	*Ornithodoros moubata*	Waxman and Connolly, 1993
	TAI	*O. moubata*	Karczewski et al., 1995
	Disaggregin	*O. moubata*	Karczewski et al., 1994
	Savignygrin	*Ornithodoros savignyi*	Mans et al., 2002b
	Monogrin	*Argas monolakensis*	Mans and Ribeiro, 2008
	Ixodegrin	*I. scapularis, I. pacificus*	Francischetti et al., 2005b
	SGE, Fraction AV 16/3	*Amblyomma variegatum*	Kazimirova et al., 2002
	Longicomin	*H. longicornis*	Cheng et al., 1999
	Variabilin	*D. variabilis*	Wang et al., 1996
***Blood coagulation***	Ornithodorin	*O. moubata*	van de Locht et al., 1996
	TAP	*O. moubata*	Waxman et al., 1990
	Enolase	*O. moubata*	Diaz-Martin et al., 2013a
	Savignin	*Ornithodoros savignyi*	Nienaber et al., 1999
	TAP-like protein	*O. savignyi*	Joubert et al., 1998
	Monobin	*Argas monolakensis*	Mans and Ribeiro, 2008
	Ixolaris	*I. scapularis*	Francischetti et al., 2002
	Penthalaris	*I. scapularis*	Francischetti et al., 2004
	Salp 14	*I. scapularis*	Narasimhan et al., 2002
	TIX-5	*I. scapularis*	Schuijt et al., 2013
	Metalloprotease	*I. scapularis*	Francischetti et al., 2003
	IxscS-1E1	*I. scapularis*	Ibelli et al., 2014
	Ir-CPI	*I. ricinus*	Decrem et al., 2009
	IRS-2	*I. ricinus*	Chmelar et al., 2011
	Americanin	*A. americanum*	Zhu et al., 1997
	AamS6	*A. americanum*	Mulenga et al., 2013a
	AamAV422	*A. americanum*	Mulenga et al., 2013b
	Serpin19	*A.americanum*	Kim et al., 2015b
	Calreticulin	*A. americanum*	Jaworski et al., 1995
	Variegin	*Amblyomma variegatum*	Koh et al., 2007
	Amblyomin-X	*Amblyomma cajennense*	Batista et al., 2010; Branco et al., 2016
	Madanin-1; Madanin-2	*H. longicornis*	Cheng et al., 1999
	Chimadanin	*H. longicornis*	Nakajima et al., 2006
	Haemaphysalin	*H. longicornis*	Kato et al., 2005
	Longistatin	*H. longicornis*	Anisuzzaman et al., 2011a
	65 kDa protein	*R. appendiculatus*	Limo et al., 1991
	Rhipilin-1; Rhipilin-2	*Rhipicephalus hemaphysaloides*	Gao et al., 2011; Cao et al., 2013
	BmAP	*Rhipicephalus (Boophilus) microplus*	Horn et al., 2000
	Microphilin	*R. (B.) microplus*	Ciprandi et al., 2006
	RmS-15	*R. (B.) microplus*	Xu et al., 2016
	Calcaratin	*Boophilus calcaratus*	Motoyashiki et al., 2003
	Hyalomin-1	*Hyalomma marginatum rufipes*	Jablonka et al., 2015
***Innate immune responses***	MIF homolog	*A. americanum*	Jaworski et al., 2001
	MIF homolog	*H. longicornis*	Umemiya et al., 2007
	Ir-LBP	*I. ricinus*	Beaufays et al., 2008
	Salp16 Iper1, Salp16 Iper2	*Ixodes persulcatus*	Hidano et al., 2014; Liu X. Y. et al., 2014
	IRS-2	*I. ricinus*	Palenikova et al., 2015
	Evasin-1; Evasin-3; Evasin 4	*Rhipicephalus sanguineus*	Frauenschuh et al., 2007; Deruaz et al., 2008
	Hyalomin A, B	*H. asiaticum*	Wu et al., 2010
	Amregulin	*A. variegatum*	Tian et al., 2016
	Ado, PGE_2_	*R. sanguineus*	Oliveira et al., 2011
	Metalloproteases	*Amblyomma maculatum*	Jelinski, 2016
	SHBP	*D. reticulatus*	Sangamnatdej et al., 2002
	RaHBP(M), RaHBP(F)	*R. appendiculatus*	Paesen et al., 1999
	TdPI	*R. appendiculatus*	Paesen et al., 2007
***Complement***	OmCI	*O. moubata*	Nunn et al., 2005
	TSGP2, TSGP3	*O. savignyi*	Mans and Ribeiro, 2008
	Isac	*I. scapularis*	Valenzuela et al., 2000
	Salp 20	*I. scapularis*	Tyson et al., 2007; Hourcade et al., 2016
	Irac I, II, Isac paralogues	*I. ricinus*	Daix et al., 2007
***Acquired immune responses***	Salp15	*I. scapularis*	Anguita et al., 2002; Ramamoorthi et al., 2005
	IL-2 binding protein	*I. scapularis*	Gillespie et al., 2001
	Sialostatin L, L2	*I. scapularis*	Kotsyfakis et al., 2006
	Iris	*I. ricinus*	Leboulle et al., 2002
	BIP	*I. ricinus*	Hannier et al., 2004
	Salp15-like protein	*I. ricinus*	Liu J. et al., 2014
	Salp15 Iper-1, Salp15 Iper-2	*I. persulcatus*	Mori et al., 2010
	P36	*D. andersoni*	Bergman et al., 2000
	Japanin	*R. appendiculatus*	Preston et al., 2013
	BIF	*Hyalomma asiaticum*	Yu et al., 2006

*tHRF, tick histamine release factor; ISL 929 and ISL 1373, I. scapularis salivary proteins 929 and 1373; HLTnI, troponin I-like molecule; PG, prostaglandin; TAI, Tick adhesion inhibitor; TAP, tick anticoagulant peptide; TIX-5, tick inhibitor of factor Xa toward factor V; IxscS-1E1, blood meal-induced I. scapularis (Ixsc) tick saliva serine protease inhibitor (serpin (S); Ir-CPI, coagulation contact phase inhibitor from I. ricinus; IRS, I. ricinus serpin; AamS6, Amblyomma americanum tick serine protease inhibitor 6; AamAV422, Amblyomma americanum AV422 protein; BmAP, Rhipicephalus (Boophilus) microplus anticoagulant protein; RmS-15, Rhipicephalus (Boophilus) microplus serpin 15; MIF, macrophage migration inhibitory factor; Ir-LBP, Ixodes ricinus salivary LTB4-binding lipocalin; Iper, I. persulcatus; Ado, adenosine; SHBP, serotonin- and histamine-binding protein; RaHBP(M), RaHBP(F), Female (F) and male (M) Rhipicephalus appendiculatus histamine-binding protein; TdPI, tick-derived peptidase inhibitor; OmCI, O. moubata complement inhibitor; TSGP2, TSGP3, soft tick lipocalins, Isac, I. scapularis salivary anticomplement; Irac, I. ricinus anticomplement; Salp, salivary protein; Iris, I. ricinus immunosuppressor; BIP, B-cell inhibitory protein; P36, 36-kDa immunosuppressant protein; BIF, B-cell inhibitory factor*.

### Impact of tick saliva on host haemostasis

Ticks have developed strategies to block different arms of the haemostatic system of their hosts, and it has been suggested that these antihaemostatic strategies have evolved independently in argasid and ixodid ticks (Mans et al., 2008; Mans, 2011). Haemostasis refers to a set of processes—including vasoconstriction, formation of a platelet plug, blood coagulation, and fibrinolysis—that together control blood loss following vascular injury and ensure normal blood flow (Hoffman et al., 2009). After vascular injury, platelets adhere to exposed subendothelial tissue and are activated, principally owing to the engagement of platelet surface receptors by von Willebrand factor and collagen. Initial activation of platelets leads to release of soluble mediators—such as ADP, serotonin and thromboxane A_2_—which activate additional platelets, and to activation of platelet integrins. Integrin binding to multiple ligands in subendothelial tissue or on the surface of other platelets promotes further activation and aggregation of platelets, ultimately leading to the formation of a platelet plug. Moreover, serotonin and thromboxane A_2_ trigger vasoconstriction.

To counteract host-derived **vasoconstrictors**, ticks secrete vasodilators into the site of tissue injury, e.g., non-proteinaceous, lipid-derived substances, such as prostacyclin and prostaglandins (see Table 1). Certain salivary proteins, such as tick histamine release factor (tHRF) from *Ixodes scapularis* (Dai et al., 2010) and the serine proteinase inhibitor (serpin) IRS-2 from *Ixodes ricinus*, the latter of which inhibits chymase and cathepsin G (Chmelar et al., 2011), may modulate vascular permeability as well (Chmelar et al., 2012). An activity that counters vasoconstriction has also been evidenced in the salivary gland extracts (SGE) of *Dermacentor reticulatus* and *Rhipicephalus appendiculatus*, and while as yet unidentified, the active molecule(s) do not appear to belong to the prostaglandin family (Pekarikova et al., 2015).

Primary haemostasis, that is, **platelet activation and aggregation** at the site of vascular injury, is targeted by ticks in various manners (Francischetti, 2010). The tick adhesion inhibitor (TAI), found in *Ornithodoros moubata*, interferes with adhesion of platelets to soluble collagen and their ensuing activation and aggregation (Waxman and Connolly, 1993; Karczewski et al., 1995). In soft ticks (Ribeiro et al., 1991; Mans et al., 1998a, 2002a), as well as in certain hard tick species (Ribeiro et al., 1985; Liyou et al., 1999), salivary apyrase [an adenosine triphosphate (ATP)-diphosphohydrolase] has been found to degrade ADP. Salivary prostaglandins, such as PGI_2_ from *I. scapularis* (Ribeiro et al., 1988) or PGF2á from *A. americanum* (Aljamali et al., 2002) may induce increase in cAMP, an intracellular platelet aggregation inhibitor (Francischetti, 2010). Moubatin, a lipocalin derived from *O. moubata*, inhibits collagen-induced platelet aggregation by scavenging thromboxane A_2_ (Waxman and Connolly, 1993; Karczewski et al., 1995; Mans and Ribeiro, 2008). Longicornin, isolated from the SG of *Haemapysalis longicornis*, also inhibits collagen-mediated platelet aggregation, by a mechanism that remains to be defined (Cheng et al., 1999).

While playing an essential role in secondary haemostasis, as discussed below, the serine protease thrombin also activates platelets through cleavage of protein-activated receptors at the platelet surface. Tick salivary antithrombins can inhibit platelet aggregation induced by thrombin (Hoffmann et al., 1991; Nienaber et al., 1999; Kazimirova et al., 2002). The IRS-2 serpin from *I. ricinus* was found to inhibit platelet aggregation induced by both thrombin and cathepsin G (Chmelar et al., 2011), and the serpin IxscS-1E1 from *I. scapularis* to inhibit thrombin- and ADP-induced platelet aggregation (Ibelli et al., 2014).

Post-activation inhibitors of platelet aggregation are known to target the platelet fibrinogen receptor. The disintegrin-like peptides savignygrin from *O. savignyi* (Mans et al., 2002b), monogrin from *Argas monolakensis* (Mans and Ribeiro, 2008), and variabilin from *D. variabilis* (Wang et al., 1996) display the integrin recognition motif RGD and prevent the binding of other ligands to the platelet receptor. By contrast, the fibrinogen receptor antagonist disaggregin, derived from *O. moubata*, lacks the RGD motif and prevents ligand binding and hence platelet aggregation by different means (Karczewski et al., 1994). Ixodegrins identified in *Ixodes pacificus* and *I. scapularis* are integrin antagonists that display sequence similarity to variabilin (Francischetti et al., 2005b; Francischetti, 2010). In addition, ticks have evolved strategies to disaggregate platelet aggregates, either by displacement of fibrinogen from its receptor by competitive binding, e.g., savignygrin (Mans et al., 2002c), or by fibrinolysis (Decrem et al., 2009; Anisuzzaman et al., 2011b; Diaz-Martin et al., 2013a).

Secondary haemostasis, which refers to **blood coagulation**, occurs concomitantly with primary haemostasis. Blood coagulation involves a series of enzymatic reactions during which a coagulation factor (inactive proenzyme) is converted to an active form, which then activates the next proenzyme. Thrombin is involved in the final (common) pathway of the blood coagulation cascade. It converts fibrinogen into fibrin and also regulates the activity of other coagulation factors. Different coagulation factors are countered by multiple tick salivary components, of which Kunitz-type proteinase inhibitors are the most abundant class (Koh and Kini, 2009; Chmelar et al., 2012). Tick-derived thrombin inhibitors target the enzyme at different sites and through various mechanisms (Koh and Kini, 2009). Ornithodorin (van de Locht et al., 1996), savignin (Nienaber et al., 1999), and monobin (Mans et al., 2008) from soft ticks are Kunitz-type antithrombins, while IRS-2 and IRIS from *I. ricinus* (Leboulle et al., 2002; Chmelar et al., 2011), IxscS-1E1 from *I. scapularis* (Ibelli et al., 2014) and RmS-15 from *Rhipicephalus (Boophilus) microplus* (Xu et al., 2016) are serpins. Antithrombins belonging to the hirudin-like/madanin/variegin superfamily include madanin 1 and 2 from *H. longicornis* (Iwanaga et al., 2003), variegin from *Amblyomma variegatum* (Koh et al., 2007) and hyalomin-1 from *Hyalomma marginatum rufipes* (Jablonka et al., 2015). The antithrombins microphilin (Ciprandi et al., 2006) and BmAP (Horn et al., 2000) from *R. (B.) microplus* and calcaratin from *Boophilus calcaratus* (Motoyashiki et al., 2003) cannot be classified in any of the previously mentioned groups.

TAP, the Kunitz-type tick anticoagulant peptide from *O. moubata* (Waxman et al., 1990), the TAP-like protein from *O. savignyi* (Joubert et al., 1998), amblyomin-X from *Amblyomma cajennense* (Batista et al., 2010) and Salp14 (a basic tail-secreted protein) from *I. scapularis* (Narasimhan et al., 2002) are all inhibitors of coagulation factor Xa. Kunitz-type inhibitors that display similarity to the tissue factor (TF) pathway inhibitor have been identified, e.g., in *I. scapularis* (Ixolaris and penthalaris) (Francischetti et al., 2002, 2004), and Rhipicephalus hemaphysaloides (Rhipilin-1 and -2) (Gao et al., 2011; Cao et al., 2013). SGE from *D. andersoni* was observed to inhibit factor V and factor VII (Gordon and Allen, 1991). TIX-5, found in SG of *I. scapularis* nymphs, was shown to inhibit the activation of factor V by factor Xa, and thus delay activation of the coagulation cascade (Schuijt et al., 2013). Haemaphysalin, a plasma kallikrein-kinin system inhibitor, and Ir-CPI, a contact phase inhibitor that impairs the intrinsic coagulation pathway, were identified in *H. longicornis* (Kato et al., 2005) and *I. ricinus* (Decrem et al., 2009), respectively. IRIS, an immunomodulatory serpin from SG of *I. ricinus*, was found to impair the contact phase-activated pathway of coagulation and prolong fibrinolysis (Prevot et al., 2006). Serpins AamS6 (Mulenga et al., 2013a), AamAV422 (Mulenga et al., 2013b), and serpin19 (Kim et al., 2015b), derived from *A. americanum*, and a 65 kDa protein from *R. appendiculatus* (Limo et al., 1991) all delay plasma clotting, although their mode of action remains to be elucidated. Tick-derived calcium-binding proteins with homology to the calreticulin (CRT) sequence may also modulate haemostasis by sequestration of calcium ions, which act as cofactors of the coagulation enzymes (Jaworski et al., 1995).

Finally, tick salivary components may display **fibrinolytic activity**. A metalloprotease mediating such activity was detected in saliva of *I. scapularis* (Francischetti et al., 2003). Longistatin from *H. longicornis*, an activator of plasminogen, was found to cause hydrolysis of fibrinogen and delay formation of the fibrin clot (Anisuzzaman et al., 2011a).

### Impact of tick saliva on host immune responses

When skin integrity is compromised, processes required to repel microbial invasion and restore the barrier function of the skin are immediately deployed. Pre-positioned sentinel cells, such as mast cells (MC), macrophages and dendritic cells (DC), are activated by particular components released from damaged skin cells or expressed by microbial organisms. Soluble mediators released by MC, such as bradykinin and histamine, cause itch and pain. Sentinel cells release chemoattractants, including chemokines and leukotrienes, that recruit blood-borne innate immune cells, such as neutrophils and monocytes, to the site of damage, as well as pro-inflammatory cytokines, e.g., tumor necrosis factor alpha (TNFα) and interleukin (IL)-1, that enhance activation of local and infiltrating innate immune cells. Monocytes secrete growth hormones that induce proliferation of fibroblasts and deposition of extracellular matrix, thus contributing to wound healing. Activated DC that have acquired foreign antigen migrate via lymphatics to skin-draining lymph nodes, where antigen may be presented to naïve B and T lymphocytes, thereby initiating an adaptive immune response that culminates in the generation of antigen-specific antibodies and T lymphocytes.

**Itch and pain**—principally triggered by MC- and basophil-derived mediators—cause awareness of injury and, if unchecked, would arouse an alleviative behavioral response. Ticks mitigate itch and pain by means of salivary components that degrade bradykinin and sequester histamine. For example, salivary metalloproteases from *I. scapularis* (Ribeiro and Mather, 1998) and *Amblyomma maculatum* (Jelinski, 2016) hydrolyse bradykinin. Amine-binding proteins of the lipocalin family, e.g., the histamine-binding proteins RaHBP(M) and RaHBP(F)-1,2 from *R. appendiculatus* (Paesen et al., 1999) and the serotonin- and histamine-binding protein SHBP from *D. reticulatus* (Sangamnatdej et al., 2002), have been found to interfere with the activity of histamine and serotonin, the latter representing an important inflammatory mediator in rodents (Askenase et al., 1980). Moreover, the activity of human β-tryptases, which are MC-specific serine proteases involved in inflammation, is diminished by the tick-derived protease inhibitor (TdPI) from *R. appendiculatus* (Paesen et al., 2007).

**Recruitment of blood-borne innate immune cells**, and notably neutrophils, is also strongly suppressed by tick saliva. Salivary inhibitors of CXCL8 and of several CC chemokines (CCL-2, -3, -5, and -11) were evidenced in SGE of several ixodid species (Hajnicka et al., 2001). Three chemokine-binding proteins, called Evasin-1, -2, and -3, were subsequently purified from the SG of *Rhipicephalus sanguineus* (Frauenschuh et al., 2007) and shown to present selectivity for different chemokines (Frauenschuh et al., 2007; Deruaz et al., 2008). Later work has suggested that Evasin-3-like activity may be common among metastriate ixodid tick species (Vancova et al., 2010a). CXCL8-mediated chemotaxis of neutrophils is also inhibited by Salp16 Iper1 and Salp16 Iper2, salivary proteins from *Ixodes persulcatus* (Hidano et al., 2014). Ir-LBP, a lipocalin from *I. ricinus*, was found to bind leukotriene B4, an important inflammatory mediator, with high affinity, thereby interfering with neutrophil chemotaxis and activation (Beaufays et al., 2008). Several tick species express a homolog of the vertebrate macrophage migration inhibitory factor (MIF) (Wasala and Jaworski, 2012). For both *A. americanum* (Jaworski et al., 2001) and *H. longicornis* (Umemiya et al., 2007), MIF has been shown to inhibit migration of macrophages in *in vitro* assays, suggesting that MIF might diminish macrophage recruitment to the bite location *in vivo*.

Tick saliva quells **inflammation** at the bite location by diminishing or enhancing secretion of pro- and anti-inflammatory cytokines, respectively. *D. andersoni* SGE diminished production of IL-1 and TNFα by macrophages and IL-2 and interferon (IFN)-γ production by T lymphocytes (Ramachandra and Wikel, 1992). Hyalomin-A and -B from SG of *Hyalomma asiaticum asiaticum* were found to inhibit secretion of TNFα, CCL2, and IFN-γ, but to increase secretion of the immunosuppressive cytokine IL-10 (Wu et al., 2010). Amregulin from *A. variegatum* saliva was found to suppress the *in vitro* production of TNFα, IL-1, CXCL8, and IFN-γ (Tian et al., 2016). Moreover, non-proteinaceous substances such as PGE_2_ and Ado (purine nucleoside adenosine) from saliva of *R. sanguineus* have been found to impair the production of the pro-inflammatory cytokines IL-12p40 and TNFα and stimulate the production of IL-10 by murine DC (Oliveira et al., 2011). Beyond its impact on cytokine production, tick saliva counters multiple effector functions of innate immune cells, including the production of reactive oxygen species (ROS) by neutrophils (Ribeiro et al., 1990; Guo et al., 2009) and macrophages (Kopecký and Kuthejlová, 1998), phagocytosis by neutrophils (Ribeiro et al., 1990) and macrophages (Kramer et al., 2011) and cytotoxicity of NK cells (Kubes et al., 1994). IRS-2, an *I. ricinus* serpin, targets cathepsin G and chymase, enzymes produced by activated neutrophils and MC, respectively (Pekarikova et al., 2015).

Tick saliva also restricts **wound healing and angiogenesis** (Francischetti, 2010; Hajnicka et al., 2011). Salivary molecules from hard ticks are able to bind to the transforming growth factor (TGF)-β1, the platelet-derived growth factor (PDGF), the fibroblast growth factor (FGF)-2 and the hepatocyte growth factor (HGF), depending on the tick species (Hajnicka et al., 2011; Slovak et al., 2014). *Dermacentor variabilis* saliva was found to suppress basal and PDGF-stimulated fibroblast migration and reduce PDGF-stimulated activity of extracellular signal-regulated kinase (ERK) (Kramer et al., 2008). Tick compounds similar to disintegrin metalloproteases and thrombospondin can impair cell-matrix interactions and angiogenesis (Valenzuela et al., 2002; Francischetti et al., 2005a; Fukumoto et al., 2006). The *I. scapularis* proteins ISL 929 and ISL 1373, for example, impair both the expression of β2 integrins and the adherence of polymorphonuclear leukocytes (PMN) (Guo et al., 2009). A troponin I-like molecule (HLTnI) (Fukumoto et al., 2006) and a Kunitz-type protein (haemangin) (Islam et al., 2009) from the SG of *H. longicornis* also impair angiogenesis and wound healing.

**The complement system** links the host innate and adaptive immune responses and is activated through three pathways (alternative, classical, and lectin). The alternative pathway is the main line of defense against invading pathogens and is also involved in resistance to ticks (Wikel, 1979). Isac, Salp20 and Isac-1 from *I. scapularis* (Valenzuela et al., 2000; Tyson et al., 2007) and IRAC I and II from *I. ricinus* (Daix et al., 2007; Couvreur et al., 2008) inhibit formation of the C3 convertase of the alternative pathway by impeding the binding of complement factor B to complement C3b. The lipocalins OmCI (*O. moubata* complement inhibitor; Nunn et al., 2005) and TSGP2 and TSGP3 (Mans and Ribeiro, 2008) specifically target C5 activation. Inhibition of the classical complement pathway has also been reported for saliva and SGE of *A. cajennense* (Franco et al., 2016).

Tick saliva or SGE is also able to suppress the initiation of **adaptive immunity**, such as by interfering with the capacity of DC to present antigen to T cells and prime appropriate Th responses (Cavassani et al., 2005; Mejri and Brossard, 2007; Oliveira et al., 2008; Skallova et al., 2008; Carvalho-Costa et al., 2015). In some instances these activities have been assigned to molecularly-defined salivary components. Salivary cysteine protease inhibitors (cystatins) of *I. scapularis* were shown to possess inhibitory activity against certain cathepsins. Both Sialostatin L and L2 strongly inhibited cathepsin L, but Sialostatin L also inhibited cathepsin S, which is involved in antigen processing. Inhibition of cathepsin S by Sialostatin L diminished the capacity of DC to induce proliferation in antigen-specific CD4^+^ T cells (Kotsyfakis et al., 2006; Sa-Nunes et al., 2009), while Sialostatin L2 suppressed the type I IFN response in DC (Lieskovska et al., 2015b). Salp15 from *I. scapularis*, upon stimulation of DC by TLR-2 and -4 ligands, suppressed the production of pro-inflammatory cytokines IL-12p70, IL-6, and TNFα, and the capacity of DC to activate T cells (Hovius et al., 2008a). Japanin, a salivary gland lipocalin from *R. appendiculatus*, modifies the expression of co-stimulatory and co-inhibitory molecules, the production of diverse cytokines, and inhibits DC differentiation from monocytes (Preston et al., 2013). Not all immunomodulatory salivary mediators are proteins: in *I. scapularis* saliva, PGE_2_ was found to be a major inhibitor of DC maturation and TLR-ligand induced secretion of IL-12 and TNFα (Sá-Nunes et al., 2007).

In a number of studies, tick saliva or SGE has been observed to enhance production of T helper 2 (Th2) signature cytokines, such as IL-4, and diminish production of Th1 cytokines, e.g., IFN-γ (Ferreira and Silva, 1999; Mejri et al., 2001). T cell inhibitory salivary molecules include a secreted IL-2 binding protein in *I. scapularis*, which suppresses T cell proliferation and other IL-2 dependent activities (Gillespie et al., 2001), P36 in *D. andersoni* (Bergman et al., 2000), Iris in *I. ricinus* (Leboulle et al., 2002), and Salp15 in *I. scapularis* (Anguita et al., 2002). Iris was found to suppress T cell proliferation, promote a Th2 type response and inhibit the production of pro-inflammatory cytokines IL-6 and TNFα. Salp15 binds to CD4 molecules on the surface of CD4^+^ T (helper) cells, and consequently inhibits signaling mediated by T cell receptors, resulting in decreased IL-2 production and T cell proliferation (Anguita et al., 2002; Garg et al., 2006). Nevertheless, transcriptional profiling of the cutaneous response in mice to primary and secondary infestation by *I. scapularis* nymphs did not evidence a predominant Th subset in primary infestation and evidenced a mixed Th1/Th2 and possibly regulatory T cell response in secondary infestation (Heinze et al., 2012a,b). For secondary infestation with *D. andersoni* nymphs, the same authors observed an indeterminant Th profile in skin, but a pronounced upregulation of IL-4 expression in draining lymph nodes, suggesting that the systemic response to repeated infestation may display a marked Th2 bias (Heinze et al., 2014).

Tick salivary components also disarm the humoral arm of the adaptive immune response. BIP and BIF derived from *I. ricinus* and *H. asiaticum asiaticum*, respectively, were found to suppress B-cell responses (Hannier et al., 2004; Yu et al., 2006). Salivary immunoglobulin (IgG)-binding proteins bind ingested host IgG and facilitate their excretion in saliva during feeding, thus protecting ticks from ingested host IgG (Wang and Nuttall, 1999).

Owing—at least in part—to active interference by salivary compounds with development of an appropriate immune response, susceptible hosts, such as mice, may fail to develop acquired resistance to ticks despite repeated tick feeding (Schoeler et al., 1999). In tick resistant hosts (e.g. guinea pigs, rabbits), however, the presence of antibodies and effector T lymphocytes with specificity for tick antigens assures a rapid secondary response to infestation that impairs tick feeding. Significant diversity in resistance to tick infestation has been also observed among different breeds of cattle, some of which is related to immunity. In particular, T-cell-mediated responses directed against larval feeding, rather than IgG responses to *R. (B.) microplus* antigens, were shown to be protective (Jonsson et al., 2014). Microarray analysis of differentially expressed genes in bovine skin early after attachment of *R. (B.) microplus* larvae revealed an important role for lipid metabolism in control of inflammation and impairment of tick infestation in tick-resistant cattle, and an impairment of the acute phase response in susceptible animals (Carvalho et al., 2014). Variation among breeds in non-immune characteristics, such as the extracellular matrix of the skin, is also likely to contribute to the variation in resistance to ticks (Jonsson et al., 2014).

## Tick saliva and pathogen transmission

Host anti-tick immunity may have a major impact on both transmission and acquisition of TBP by ticks. For example, repeated infestation of resistant strains of laboratory animals with pathogen-free *I. scapularis* nymphs afforded protection against tick-transmitted *B. burgdorferi*, suggesting that immunity against tick salivary antigens can impair transmission of *Borrelia* (Wikel et al., 1997; Nazario et al., 1998). Moreover, immunization of guinea pigs with *I. scapularis* SG proteins produced during the first day of tick feeding interfered with *Borrelia* transmission from ticks to hosts, suggesting that at least some of the antigens involved in acquired resistance are secreted in saliva during the first 24 h of tick attachment (Narasimhan et al., 2007a). Indeed, the aforementioned Th2 polarization of the immune response by salivary immunomodulatory molecules has been proposed to enhance transmission of TBP (Gillespie et al., 2000; Schoeler and Wikel, 2001; Wikel and Alarcon-Chaidez, 2001; Brossard and Wikel, 2008).

Enhancement of pathogen transmission by tick saliva—called saliva-assisted transmission or SAT—has been documented for several tick-pathogen associations (Nuttall and Labuda, 2004). However, relatively few tick molecules implicated in pathogen transmission have been identified and characterized.

### Saliva assisted transmission

In the course of co-evolution with ticks and vertebrate hosts, tick-borne microorganisms have developed myriad strategies to subvert tick salivary molecules so as to ensure their transmission cycle (Brossard and Wikel, 2004; Nuttall and Labuda, 2004; Ramamoorthi et al., 2005; Wikel, 2013). Saliva not only provides the matrix in which TBP are inoculated, but also profoundly modifies the local environment at the bite location, with consequences for not only transmission of TBP from infected ticks to the uninfected vertebrate host but also acquisition of TBP by uninfected ticks.

The first observation of SAT concerned Thogoto virus (THOV). Acquisition of infection by virus-free *R. appendiculatus* nymphs was enhanced when they fed on guinea pigs injected with a mixture of THOV and SGE (Jones et al., 1989). Subsequent studies have demonstrated the SAT phenomenon for tick-borne encephalitis virus (TBEV) (Alekseev and Chunikhin, 1990; Labuda et al., 1993) and *B. burgdorferi* s.l. (Gern et al., 1993; Pechova et al., 2002; Zeidner et al., 2002; Machackova et al., 2006). Tick saliva has also been shown to enhance infection of vertebrate hosts, as documented for Powassan virus (Hermance and Thangamani, 2015), African swine fever virus (ASFV) (Bernard J. et al., 2016), *B. burgdorferi* s.l. (Pechova et al., 2002; Zeidner et al., 2002; Machackova et al., 2006), *Francisella tularensis* (Krocova et al., 2003), and *Rickettsia conorii* (Milhano et al., 2015).

As a corollary of SAT, saliva is also presumed to enhance so-called non-viraemic transmission (NVT), i.e., transmission of TBP from infected to pathogen-free ticks that feed concomitantly on the same host, in the absence of systemic infection of the host. The pool of saliva produced by ticks that feed in close proximity is thought to enhance pathogen exchange between co-feeding ticks (Randolph, 2011; Voordouw, 2015). Indeed, transmission of THOV to uninfected *R. appendiculatus* ticks was observed to be more efficient while co-feeding with infected ticks on non-viraemic guinea pigs than while feeding on viraemic hamsters (Jones et al., 1987). NVT may assist TBP in circumventing the host immune response, such as by permitting viruses to evade virus-specific neutralizing antibodies (Labuda et al., 1997). Moreover, during NVT of TBEV between *I. ricinus* ticks, infectious virus was evidenced in the cellular infiltrate at the tick bite location, suggesting that migratory cells might transport the virus from infected to uninfected co-feeding ticks (Labuda et al., 1996). While NVT is thought to be most efficient for viruses, it has also been observed for various combinations of tick-borne bacteria and ticks, including *B. burgdorferi* s.l. and *Ixodes* spp (Gern and Rais, 1996; Piesman and Happ, 2001; Richter et al., 2002), *R. conorii* and *R. sanguineus* (Zemtsova et al., 2010). *Rickettsia parkeri* and *A. maculatum* (Banajee et al., 2015) or the *Ehrlichia muris*-like agent and *I. scapularis* (Karpathy et al., 2016).

In most instances SAT has been attributed to the immunomodulatory activity of salivary compounds, a paradigm that has been most extensively addressed for tick-borne bacteria, and more particularly *B. burgdorferi*. Indeed, when mice were infected with *B. burgdorferi* by syringe inoculation they developed a robust humoral response against the protective surface antigens Osp-A and -B, but failed to do so when infected by a tick bite (Gern et al., 1993). Moreover, BALB/c mice have been reported to develop a Th2-biased immune response against *B. burgdorferi* following an infectious tick bite, but a mixed Th1/Th2 response after injection (Christe et al., 2000). Furthermore, when *Borrelia* was injected into mice along with *I. ricinus* saliva, the number of leukocytes and T lymphocytes in the epidermis was diminished at early time-points after inoculation and the total number of cells in draining lymph nodes was reduced (Severinova et al., 2005). For *Borrelia* sp., the mechanisms that underlie SAT have been extensively addressed in relation to DC (for review see (Mason et al., 2014)). *In vitro* treatment of murine DC with *I. ricinus* saliva was found to inhibit DC maturation (Skallova et al., 2008), reduce phagocytosis of *B. afzelii*, reduce cytokine production, and impair proliferation and IL-2 production in *Borrelia*-specific CD4^+^ T cells (Slamova et al., 2011). Moreover, *I. ricinus* saliva modulated IFN-γ signaling pathways in DC (Lieskovska and Kopecky, 2012b), as well as pathways activated by a TLR2 ligand in *Borrelia*-stimulated DC (Lieskovska and Kopecky, 2012a). SGE of *I. ricinus* and saliva of *I. scapularis* inhibited *in vitro* killing of *Borrelia* by macrophages, through reduced production of ROS (Kuthejlova et al., 2001), and PMN (Montgomery et al., 2004), respectively. In the skin, expression of an anti-microbial peptide (AMP), cathelicidin, was induced by syringe inoculation of *Borrelia* but markedly suppressed when introduced by *I. ricinus* (Kern et al., 2011). Moreover, SGE derived from *I. ricinus* inhibited the *in vitro* inflammatory response of human primary keratinocytes induced by *Borrelia* or by OspC, a major surface antigen. In particular, chemokines (CXCL8 and CCL2) and AMPs (defensins, cathelicidin, psoriasin, and RNase 7) were down-regulated (Marchal et al., 2011). *I. ricinus* saliva also inhibited cytokine production by human primary keratinocytes in response to TLR2/TLR3 ligands during *Borrelia* transmission (Bernard Q. et al., 2016). Nevertheless, the effects of *I. scapularis* saliva on resident skin cells exposed to *Borrelia* were found to depend on the cell type (Scholl et al., 2016): tick saliva suppressed the production of the pro-inflammatory mediators IL-6, CXCL8, and TNFα by monocytes, but enhanced the production of CXCL8 and IL-6 by dermal fibroblasts.

Salivary mediators also appear to influence transmission of Rikettsiales bacteria. Saliva of *I. scapularis* was found to inhibit the pro-inflammatory cytokine response of murine macrophages to infection with the intracellular bacterium, *Anaplasma phagocytophilum* (Chen et al., 2012). For spotted group fever rickettsiae, immunomodulatory factors introduced during feeding of *A. maculatum* seemed to enhance the pathogenicity and dissemination of *R. parkeri* in rhesus macaques (Banajee et al., 2015). Mice experimentally inoculated with *R. conorii* and infested with *R. sanguineus* presented reduced levels of IL-1β and the transcription factor NF-κB and enhanced levels of IL-10 in the lung in comparison with mice inoculated with *R. conori* alone, suggesting that inflammation was inhibited (Milhano et al., 2015).

The mechanisms underlying SAT have less frequently been explored for viruses. Nevertheless, treatment of DC with *I. ricinus* saliva increased the proportion of TBEV-infected cells and decreased the production of TNFα and IL-6 and the induction of apoptosis elicited by the virus (Fialova et al., 2010).

### Tick salivary molecules implicated in pathogen transmission

The underlying molecular mechanisms implicated in TBP transmission have only begun to be elucidated, and only a few tick molecules directly or indirectly -through interaction with the host- involved in transmission, have been actually identified and functionally characterized (Table 2) (Nuttall and Labuda, 2004; Ramamoorthi et al., 2005; Kazimirova and Stibraniova, 2013; Wikel, 2013; Liu and Bonnet, 2014). Among these, some salivary compounds affect the acquisition of TBP by the vector, while others enhance the transmission of TBP to the host.

**Table 2:** Tick salivary molecules implicated in transmission of tick-borne microorganisms.

**Tick species**	**Salivary molecule**		**Microorganism**	**References**
*I. ricinus*	Calreticulin	Calcium-binding protein	*B. burgdorferi* s.l.	Cotté et al., 2014
*I. scapularis*	5.3-kD protein	Antimicrobial peptide	*A. phagocytophilum*	Liu et al., 2012
*I. scapularis*	Salp15	Secreted salivary protein	*B. burgdorferi*	Ramamoorthi et al., 2005; Schuijt et al., 2008
*I. ricinus*	Salp15 Iric-1	Secreted salivary protein, Salp15 homolog	*B. burgdorferi*	Hovius et al., 2008b
*I. persulcatus*	IperSalp15	Secreted salivary protein, Salp15 homolog	*B. burgdorferi*	Murase et al., 2015
*I. scapularis*	TSLPI	Tick salivary lectin pathway inhibitor	*B. burgdorferi*	Schuijt et al., 2011
*I. scapularis*	Salp20	Salivary anti-complement protein	*B. burgdorferi*	Tyson et al., 2007
*I. scapularis*	Isac	Salivary anti-complement protein	*B. burgdorferi*	Valenzuela et al., 2000
*I. ricinus*	IRAC I, II	Salivary anti-complement proteins	*B. burgdorferi*	Daix et al., 2007
*I. ricinus*	BIP	B-cell inhibitor	*B. burgdorferi*	Hannier et al., 2003
*I. scapularis*	Salp25D	Salivary protein, antioxidant	*B. burgdorferi*	Narasimhan et al., 2007b
*I. scapularis*	tHRF	Tick histamine release factor	*B. burgdorferi*	Dai et al., 2010
*I. ricinus*	IrSPI	Serine protease inhibitor	*B. henselae*	Liu X. Y. et al., 2014
*I. scapularis*	Salp16	Salivary gland protein	*A. phagocytophilum*	Sukumaran et al., 2006
*I. scapularis*	P11	Salivary gland protein	*A. phagocytophilum*	Liu et al., 2011
*I. scapularis*	Sialostatin L2	Salivary cysteine protease inhibitor	TBEV	Lieskovska et al., 2015b
*I. scapularis*	Sialostatins L, L2	Salivary cysteine protease inhibitors	*B. burgdorferi*	Lieskovska et al., 2015a
*I. scapularis*	Sialostatin L2	Salivary cysteine protease inhibitors	*A. phagocytophilum*	Chen et al., 2014
*I. scapularis*	subolesin	Tick protective antigen	*B. burgdorferi*	Bensaci et al., 2012
*D. variabilis*	subolesin	Tick protective antigen	*A. marginale*	Zivkovic et al., 2010

Expression of members of the 5.3-kDa family of salivary peptides that possess anti-microbial properties (Pichu et al., 2009; Liu et al., 2012) was found to be upregulated in SG of *I. scapularis* during infection with Langat virus (McNally et al., 2012), *B. burgdorferi* (Ribeiro et al., 2006), and *A. phagocytophilum* (Liu et al., 2012). RNAi knockdown of one member of the 5.3-kDa antimicrobial peptide family, encoded by gene-15, increased *A. phagocytophilum* burden in SG of *I. scapularis* and in blood of mice on which gene-15-deficient ticks fed (Liu et al., 2012). Thus, 5.3-kDa antimicrobial peptides are probably able to inhibit both acquisition and transmission of TBP. Moreover, the Janus kinase signaling transducer activator of transcription (JAK-STAT) pathway was implicated in the control of *A. phagocytophilum* infection in ticks by regulating the expression of antimicrobial peptides (Liu et al., 2012).

Distinct tick proteins promote the transmission of *B. burgdorferi* s.l. at different phases of infection, in relation to the phenotypic plasticity of this TBP (Radolf et al., 2012). In the infected tick, *Borrelia* spirochetes express outer surface protein OspA and bind to the midgut wall by means of a tick midgut protein (TROSPA) (Pal et al., 2004). Under the stimulus of a new blood meal and following tick attachment, spirochetes begin to express OspC and move from the midgut through the haemolymph to the SG, where they encounter Salp15. The secreted salivary protein Salp15, considered to be the first tick mediator of SAT to be discovered, was first identified in the SG of *I. scapularis*. Salp15 has been shown to bind to mammalian CD4 (Garg et al., 2006), inhibit activation of CD4^+^ T lymphocytes (Anguita et al., 2002), impair DC function by inhibiting TLR- and *B. burgdorferi*-induced production of pro-inflammatory cytokines by DCs, as well as DC-induced T cell activation (Hovius et al., 2008a). A Salp15 ortholog from *I. ricinus* inhibited the inflammatory response of human primary keratinocytes during transmission of *Borrelia* (Marchal et al., 2011). The activity of Salp15 has been shown to be critically important in pathogen transmission: RNAi silencing of Salp15 drastically reduced the capacity of ticks to transmit spirochetes to mice (Ramamoorthi et al., 2005), and immunization of mice with Salp15 afforded significant protection from *I. scapularis*-transmitted *B. burgdorferi* (Dai et al., 2009). In the tick SG, spirochetes bind to Salp15 via OspC, which protects them from antibody- and complement-mediated killing (Schuijt et al., 2008) and promotes their transmission and replication in the host skin (Ramamoorthi et al., 2005). Salp15 homologs Salp15 Iric-1 and IperSalp15 have been identified in *I. ricinus* (Hovius et al., 2008b) and *I. persulcatus* (Murase et al., 2015), respectively. These proteins have functions similar to those of Salp15 and appear to protect *B. burgdorferi* s.s., *B. garinii*, and *B. afzelii* from antibody-mediated killing in the host.

To evade complement-mediated killing, *Borrelia* also benefits from tick salivary proteins that inhibit complement activation at the tick bite location (de Taeye et al., 2013). The tick salivary lectin pathway (TSLP) inhibitor from SG of *I. scapularis* was found to interfere with the human lectin complement cascade and impair neutrophil phagocytosis and chemotaxis, thereby protecting *Borrelia* from killing by the lectin complement pathway (Schuijt et al., 2011). Moreover, silencing of TSLPI in *Borrelia*-infected ticks impaired experimental transmission of the spirochetes to mice. Salp20, a member of the *I. scapularis* anti-complement protein-like family of tick salivary proteins, inhibits the alternative complement pathway by binding properdin and causing dissociation of the C3 convertase (Tyson et al., 2007; Hourcade et al., 2016). Salp20 partially protects *B. burgdorferi* from lysis by normal human serum, suggesting that, together with the plasma activation factor H, Salp20 may protect *Borrelia* from components of the host complement system. Anti-complement proteins belonging to the Isac-like family, e.g., Isac from *I. scapularis* (Valenzuela et al., 2000) and its homologs IRAC I and II from *I. ricinus* (Daix et al., 2007) function very similarly to the previously described Salp20, but display different capacities to inhibit the alternative complement pathway depending upon the host species (Schroeder et al., 2007; Couvreur et al., 2008). Thus, proteins of the Isac-like family may potentially promote transmission of *Borrelia* to the vertebrate host (de Taeye et al., 2013).

BIP from *I. ricinus* SG was found to suppress B lymphocyte proliferation induced by the *B. burgdorferi* OspC, suggesting that BIP may enhance *Borrelia* transmission to the host (Hannier et al., 2003).

Salp25D is an immunodominant antioxidant salivary protein from *I. scapularis*. Silencing of Salp25D expression in SG impaired acquisition of *B. burgdorferi*. The protein is probably involved in acquisition of *B. burgdorferi* by ticks and acts as an antioxidant that promotes pathogen survival in the tick (Das et al., 2001; Narasimhan et al., 2007b).

Salivary tHRF has been shown capable of binding mammalian basophils and triggering release of histamine (Mulenga et al., 2003; Dai et al., 2010). Mulenga et al. (2003) proposed that tHRF was required for vasodilation during the rapid feeding phase of *D. variabilis*, when large volumes of blood are required. tHRF is up-regulated in *I. scapularis* SG during the rapid feeding phase and, by virtue of increasing blood flow to the tick bite location, may facilitate not only tick engorgement but also *B. burgdorferi* infection (Dai et al., 2010). Immunization of mice with the recombinant protein as well as silencing tHRF impaired tick feeding and decreased *Borrelia* burden in the host at 7 days after infection in skin and at 3 weeks in heart and joints (Dai et al., 2010). While reduced bacterial burden in mice may simply have been secondary to reduced burden in ticks, the authors suggested that the vasodilatory activity of histamine might also enhance systemic dissemination of *Borrelia* from the bite site (Dai et al., 2010).

IrSPI, a protein from *I. ricinus* SG belonging to the BPTI/Kunitz family of serine protease inhibitors, probably impairs host haemostasis and facilitates tick feeding and *Bartonella henselae* transmission, as RNAi silencing impaired tick feeding and diminished *B. henselae* load in the tick SG (Liu X. Y. et al., 2014).

Infection with *A. phagocytophilum* was reported to induce expression of the Salp16 gene in *I. scapularis* SG during feeding. Silencing of Salp16 gene expression interfered with trafficking of the bacteria ingested via the blood meal and infection of the tick SG, which demonstrated its role in persistence of this TBP within the tick (Sukumaran et al., 2006).

Silencing of P11, a secreted *I. scapularis* SG protein that was found to be upregulated in *A. phagocytophilum*-infected ticks, demonstrated that the protein enables infection of tick haemocytes and thus facilitates pathogen dissemination in the tick and its migration from the midgut to the SG through the haemolymph (Liu et al., 2011).

Sialostatin L and L2 from *I. scapularis* are potentially implicated in transmission of tick-borne viruses and bacteria. Sialostatin L2 was found to interfere with IFN-γ mediated immune responses in mouse splenic DC, resulting in enhanced replication of TBEV in DC (Lieskovska et al., 2015b) Sialostatin L and L2 modulated responses of murine bone marrow-derived DC exposed to *Borrelia* (Lieskovska et al., 2015a). Moreover, Sialostatin L2 stimulated proliferation of *B. burgdorferi* in murine skin (Kotsyfakis et al., 2010), possibly in relation to inhibition of the type I IFN response in DC (Lieskovska et al., 2015a,b). In addition, Sialostatin L2 inhibited inflammasome formation in mice during *A. phagocytophilum* infection and targeted caspase-1 activity (Chen et al., 2014).

Subolesin is a tick protective antigen with similarity to akirins, an evolutionarily conserved group of proteins in insects and vertebrates, and is suggested to control nuclear factor-kappa B (NF-kB)-dependent and independent gene expression and to play a role in tick immune responses to pathogens (Almazan et al., 2003; Naranjo et al., 2013). Upregulation of subolesin expression was observed in SG of *D. variabilis* ticks infected with *A. marginale* (Zivkovic et al., 2010). The impact of subolesin on transmission encompasses multiple vector-pathogen associations, as both gene silencing or immunization of hosts with recombinant subolesin protein resulted in decreased *A. marginale, A. phagocytophilum*, and *Babesia bigemina* burdens in their respective tick vectors (de la Fuente et al., 2006, 2010a; Merino et al., 2011). Moreover, vaccination of mice with vaccinia virus-expressed subolesin impaired engorgement of *I. scapularis* larvae and reduced acquisition of *B. burgdorferi* by tick larvae from infected mice and *Borrelia* transmission to uninfected mice (Bensaci et al., 2012). These findings suggest that subolesin may be involved in tick innate immunity to microbial agents by reducing their burden in SG while up-regulating factors facilitating pathogen acquisition by ticks.

Finally, a calcium-binding protein (CRT) has been found in the saliva of *A. americanum* and *D. variabilis* (Jaworski et al., 1995), in *R. microplus* (Ferreira et al., 2002) and *I. ricinus* (Cotté et al., 2014). Expression of the gene encoding CRT is up-regulated upon infection of *R. annulatus* with *B. bigemina* (Antunes et al., 2012) and CRT itself is up-regulated in the salivary proteome of *I. ricinus* upon infection with *B. burgdorferi* (Cotté et al., 2014). After immunization of rabbits with a fusion protein comprising the CRT of *A. americanum*, infestation with this tick caused necrotic feeding lesions (Jaworski et al., 1995). While salivary CRT might conceivably facilitate tick feeding and pathogen transmission through inhibition of thrombosis and complement, the precise role of CRT remains enigmatic. Indeed, while the CRT of *A. americanum* is able to bind to C1q, the first component of the classical complement pathway, it was not shown to inhibit activation of the complement cascade (Kim et al., 2015a).

## Impact of TBP infection on gene expression in tick SG

Similar to vertebrates, ticks are protected against invading microorganisms by an innate immune system (Hajdusek et al., 2013; Hynes, 2014). During co-evolution, tick-borne microorganisms have developed various strategies to evade or suppress the immune responses of their vectors in order to ensure survival, persistence and transmission. As a countermeasure, ticks have developed means of maintaining pathogen burden at a level that preserves fitness and further development (Smith and Pal, 2014). Indeed, several studies have provided evidence that acquisition of TBP can exert a profound effect on gene expression in various tick organs, including SG. Multiple families of genes have been shown to be regulated in tick SG following infection, but in most cases, their precise role is unknown (Liu and Bonnet, 2014; Chmelar et al., 2016a).

The impact of infection on the SG transcriptome has been addressed for several species of ticks and TBP, including *D. variabilis* and *Rickettsia montanensis* (Macaluso et al., 2003), *R. appendiculatus* and *Theileria parva* (Nene et al., 2004), *R. microplus* and *A. marginale* (Zivkovic et al., 2010; Mercado-Curiel et al., 2011; Bifano et al., 2014), *I. scapularis* and *A. phagocytophilum* (Ayllon et al., 2015; Cabezas-Cruz et al., 2016, 2017) or Langat virus (McNally et al., 2012) and *I. ricinus* and *B. henselae* (Liu X. Y. et al., 2014) or *B. afzelii* (Valdes et al., 2016). Diverse technologies have been applied to the identification of differentially expressed genes, from seminal studies based on differential-display PCR (Macaluso et al., 2003) and sequencing of expressed sequence tags (Nene et al., 2004), to later studies that applied suppression subtractive hybridization (Zivkovic et al., 2010) and microarrays (Mercado-Curiel et al., 2011; McNally et al., 2012) and to recent studies that have exploited next-generation sequencing (Liu X. Y. et al., 2014; Ayllon et al., 2015). Moreover, the impact of infection on the proteome of the SG, or of saliva itself, has been investigated for infection of *I. scapularis* with *A. phagocytophilum* (Ayllon et al., 2015; Villar et al., 2016) or *B. burgdorferi* (Dai et al., 2009) and *I. ricinus* with *B. burgdorferi* (Cotté et al., 2014). Recently, a novel multidisciplinary approach involving structural analysis of the SG transcriptome and biophysical simulations was used to predict substrate specificity for uncharacterized lipocalins in the SG of *I. ricinus* that might play a role in transmission of *B. afzelii* (Valdes et al., 2016). Depending on the study, relatively modest or extensive modulation of gene expression has been observed following SG infection, possibly reflecting not only the experimental strategy employed, but also the type of relationship established by the tick/pathogen pair in question. It has, for example, been suggested that pathogens that are highly adapted to their tick, such as *A. marginale* for *R. microplus*, and whose presence imposes a minimal fitness cost, have little effect on the SG transcriptome (Zivkovic et al., 2010; Mercado-Curiel et al., 2011), whereas pathogens that have a dramatic effect on tick fitness, such as *B. bovis* for *Rhipicephalus annulatus* (Ouhelli et al., 1987), have a greater impact.

## Tick saliva antigens for epidemiology and control

Anti-tick immunity was first described in the middle of the twentieth century (Trager, 1939), and many tick salivary proteins have since been shown to be immunogenic in vertebrate hosts (Wikel, 1996). Indeed, during feeding ticks inject multiple salivary proteins that elicit antibody responses, and it has been suggested, from the 1990s, that such responses could be used as biomarkers of host exposure to tick bites (Schwartz et al., 1990). Nevertheless, surveillance of TBD has largely relied on detection of host antibody responses to TBP or of TBP themselves in ticks or host, or on modeling/forecasting approaches (Hai et al., 2014), and evaluation of exposure to tick bites has only rarely been addressed. Biomarkers of exposure to ticks would be useful not only to assess host/vector contact, such as to evaluate the efficacy of anti-vector measures, but also to instruct diagnosis of TBD, by enabling documentation of an antecedent tick bite in patients for whom TBD is suspected. It is, in fact, well-known that self-reported tick exposure is a poor correlate of true tick exposure. The greatest challenge in this endeavor lies in identification of antigenic markers that allow discrimination among the different tick species to which the host has been exposed. Such specificity is particularly critical in areas with a high diversity of hematophagous arthropod species (Fontaine et al., 2011). Indeed, when antibodies against sonicated *Ixodes dammini* SG were first evaluated as markers of tick exposure in humans, cross-reactivity with other arthropods was shown to limit their epidemiologic utility (Schwartz et al., 1990). Since then, the use of a recombinant form of CRT has displayed higher specificity, albeit lower sensitivity, than whole SG for evaluation of exposure to *I. scapularis* bites (Sanders et al., 1999). Antibodies against CRT were thus sought in a longitudinal study that addressed the impact of educational interventions on tick exposure (Malouin et al., 2003), and further studies demonstrated that, in humans, such antibodies were found to persist for as long as a year and a half following tick exposure (Alarcon-Chaidez et al., 2006). Cross-reactivity, however, has been reported between recombinant CRT from *I. scapularis* and *A. americanum* (Sanders et al., 1998b), underscoring the challenge of developing specific ELISA tests. Efforts are thus still in progress to identify discriminant antigenic proteins in tick saliva that could be used to evaluate exposure to different tick species (Sanders et al., 1998a; Vu Hai et al., 2013a,b).

The production of antigen-specific antibodies directed against multiple tick salivary proteins (Vu Hai et al., 2013b), as well as the observation that vertebrates repeatedly exposed to tick bites develop immunity (Brossard and Wikel, 1997; Wikel and Alarcon-Chaidez, 2001) that affords a measure of protection against TBD (Bell et al., 1979; Wikel et al., 1997; Burke et al., 2005; Krause et al., 2009), has sparked interest in development of vaccinal strategies against tick bites. In contrast with conventional control measures, deployment of an anti-tick vaccine would not contaminate the environment and foodstuffs, nor have a deleterious impact on off-target species. Moreover, in light of the transmission of multiple TBP by the same tick species, vaccine strategies that target conserved processes in vector capacity would be expected to afford broad protection against multiple TBD transmitted by the same vector (Willadsen, 2004; Nuttall et al., 2006). Of note, such strategies hold the promise of protecting against uncharacterized TBP currently in circulation or as yet to emerge. The anti-tick vaccine approach is, moreover, compatible with the inclusion of multiple antigens, including those from TBP, so as to reinforce protection, and as demonstrated in the *Borrelia* sp.*/I. scapularis* infection model for OsPA and Salp15 (Dai et al., 2009). The only ectoparasite vaccines that are commercially available (in Australia and Cuba) target Bm86, a midgut protein of *R. microplus*—a one-host tick species—and interfere with feeding and subsequent egg production (Willadsen et al., 1995). Diminution of transmission was thus secondary to reduction in the population of *R. microplus* ticks. Unfortunately, the efficacy of these vaccines is geographically variable due to species and strain specificity and they are no longer commercialized in Australia. Even if orthologs of Bm86 existed in other tick species, however, the strategy was not transposable to tick species that feed on multiple hosts, such as wildlife species, in addition to the host species for which the vaccine is intended. For TBP transmitted by ticks with a broad range of hosts, vaccines that exert a direct effect on tick blood meal acquisition or vector competence must be found. Nevertheless, the anti-Bm86 vaccines provide a powerful proof of principle for the feasibility of creating and deploying anti-tick vaccines.

Salivary proteins represent good vaccine candidates as, owing to their contact with the host immune system, they may allow natural boosting of the host response upon exposure to ticks, limiting the need for repeated administrations (Nuttall et al., 2006). Targeting salivary proteins playing a key role in tick feeding is expected to interfere with completion of the blood meal and subsequently affect their reproductive fitness. Moreover, this strategy may also cause tick rejection and thus abolish or limit pathogen transmission, which typically occurs many hours or even days after tick attachment for hard ticks. Indeed, encouraging results have been obtained for several tick species including *H. longicornis* (Mulenga et al., 1999; Imamura et al., 2005; Zhang et al., 2011; Anisuzzaman et al., 2012), *R. appendiculatus* (Imamura et al., 2006), *O. moubata* and *O. erraticus* (Astigarraga et al., 1995; Garcia-Varas et al., 2010), *A. americanum* (de la Fuente et al., 2010b), *R. (B) microplus* (Andreotti et al., 2002; Merino et al., 2013; Ali et al., 2015), and *I. ricinus* (Prevot et al., 2007; Decrem et al., 2008b). Finally, anti-tick vaccination may also target salivary proteins directly implicated in TBP transmission. Studies carried out to this end concern *I. scapularis* and the salivary antigens Salp15 (Dai et al., 2009), TSLPI (Schuijt et al., 2011), and tHRF (Dai et al., 2010), as regards the transmission of *Borrelia* sp. to mice, and the salivary antigen Salp25D as regards acquisition of *Borrelia* sp. by ticks (Wagemakers et al., 2016). Last, vaccination against the cement protein 64TRP of *I. ricinus* also afforded protection against TBE transmission to mice (Labuda et al., 2006).

## Conclusion

The rapid evolution of tick distribution and density has created an urgent need for more effective methods for tick control and for surveillance and risk assessment for TBD (Heyman et al., 2010; Leger et al., 2013; Medlock et al., 2013). The deployment of anti-tick vaccines designed to reduce tick populations and/or transmission of TBP and reduce reliance on acaricides and repellents would represent a major improvement over current control measures, being environmentally safe and less likely than acaricides to select resistant strains. Risk assessment for human and animal populations will determine public and veterinary public health priorities, and instruct the implementation of appropriate countermeasures or complementary studies. These goals, however, require a better understanding of tick biology and of the tripartite relationship between ticks, TBP and vertebrate hosts, including the molecular interactions underlying TBP transmission. This includes the subversion of the host response mediated by saliva introduced into the host during tick feeding. Given the central role of tick SG and tick saliva both in tick biology and TBP transmission, their investigation may underpin the discovery of immunological markers for meaningful assessment of exposure to tick bites and as vaccinal candidates to protect against TBD. Ultimately, deciphering the physiology of the essential organ represented by tick SG may lead to the conception of hitherto unimagined strategies for controlling ticks and TBD.

## Author contributions

LS, MK, JR, and SB conducted the literature research, wrote the paper and prepared the figures and tables. All authors provided critical review and revisions.

### Conflict of interest statement

The authors declare that the research was conducted in the absence of any commercial or financial relationships that could be construed as a potential conflict of interest.
